# Anaesthetics

**Published:** 1859-02

**Authors:** 


### ANÆSTHETICS.

We quote the following article from the *Lancet*, for October 16, 1858, and have no doubt that all it asserts is true. Indeed, we have looked over the late Dr. Snow's work with much interest, and can heartily join in the recommendation of the *Lancet*, to give this "valuable monograph" a "thoughtful perusal." Notwithstanding all that is said, however, even by the highest authority, we believe the time is not far distant when chloroform will be abandoned as an anæsthetic agent. We again ask, why use an article of such uncontrollable power, when we have an equally reliable one in ether, and one which is entirely safe? It is notorious, that under the best conditions, and in the most careful hands, chloroform is a dangerous weapon. It is only lately that Mr. Erichsen, high surgical authority, has stated, both publicly and privately, that he uses chloroform as he would take an *express train*, ignoring, or risking, the danger of extra velocity. Now, is it not better to travel on the anæsthetic track a little more slowly, and be safe in our journey? Mr. Erichson himself intimates as much as this; and the evidence of every day only confirms us in the opinion we have expressed.

"The frequent recurrence of death from the administration of chloroform during the last few weeks, has induced a feeling of uneasiness and distrust of this agent both in the profession and out of it. It is very certain, however, that a great part of this feeling might very advantageously be transferred to the mode of administration which is still in vogue. It must not be forgotten that, in all these cases, the chloroform was administered loosely on a handkerchief, and in more than one instance, as has been proved, it was of a thickly woven texture, which did not allow of a free passage of air. This mode of administration is, we think, most unjustifiable. The experiment of the late Dr. Snow conclusively showed the necessity of carefully regulating the proportion of vapor in the air inspired. We strongly recommend to thoughtful perusal the valuable monograph on the subject of Anæsthesia which he has bequeathed to the profession. No one can rise from reading this valuable digest of a wide experience and the observation of ten years of scientific and practical labor, without a feeling of regret that so much carelessness should still prevail in the administration of this most potent vapor, and a sense of the necessity of a more extended instruction in the principles of anæsthetization. We commend the following sentences to the very careful consideration of surgeons:—

"The great point to be observed in causing insensibility by any narcotic vapor, is to present to the patient such a mixture of vapor and air as will produce its effects gradually, and enable the medical man to stop at the right moment. Insensibility is not caused so much by giving a dose as by performing a process. Nature supplies but one mixture of diluted oxygen, from which each creature draws as much as it requires; and so, in causing narcotism by inhalation, if a proper mixture of air and vapor is supplied, each patient will gradually inhale the requisite quantity of the latter to cause insensibility, according to his size and strength. It is, indeed, desirable to vary the proportions of vapor and air, but rather according to the purpose one has in view, whether medical, obstetric or surgical, than on account of the age or strength of the patient; for the respiratory process bears such a relation to the latter circumstance, as to cause each person to draw his own proper dose from a similar atmosphore in a suitable time.'

"The proportion of chloroform most suitable to produce insensibility, Dr. Snow found to be about four cubic inches of vapor, or rather more than five grains of chloroform to one hundred cubic inches of air. With a properly arranged inhaler, it is easily possible to supply the vapor in this fixed position. This simple precaution would rob chloroform of nearly all its terrors and its dangers. It is a very surprising consideration, that while the niceties of surgical manipulation are invariably attended to with the utmost care, the production of anæsthesia is very frequently left in the hands of quite inexperienced persons. It is sad to see the issues of life and death treated with indifference; it would be yet more so, if we were not well aware that the fault is one of thoughtlessness, and that those who have fallen into it will probably shrink from a similar error now that it has been pointed out so forcibly by recent events."

